# Evaluation of venous thromboembolism (VTE) risk assessment and thrombo-prophylaxis practices in hospitalized medical and surgical patients at Aga Khan Hospital Dar es Salaam: single-centre retrospective study

**DOI:** 10.11604/pamj.2022.42.160.28278

**Published:** 2022-06-29

**Authors:** Allyzain Ismail, Nikhil Jadawji, Philip Adebayo, Ahmed Jusabani, Kamran Hameed, Ali Akbar Zehri, Willy Kiviri, Athar Ali

**Affiliations:** 1 Department of Surgery, Aga Khan Hospital, Dar es Salaam, Tanzania; 2 Department of Pharmacy, Aga Khan Hospital, Dar es Salaam, Tanzania; 3 Department of Medicine, Aga Khan Hospital, Dar es Salaam, Tanzania; 4 Department of Radiology, Aga Khan Hospital, Dar es Salaam, Tanzania; 5 Department of Anesthesia, Aga Khan Hospital, Dar es Salaam, Tanzania

**Keywords:** Risk assessment, thromboprophylaxis, venous thromboembolism, retrospective study, hospitalization

## Abstract

**Introduction:**

venous thromboembolism is a complication among admitted medical and surgical patients. International guidelines recommend patients are assessed upon admission and appropriate thromboprophylaxis should be initiated. However, studies have shown that thromboprophylaxis for patients at risk of venous thromboembolism is underutilized.

**Methods:**

this was a retrospective study conducted on hospitalized medical and surgical patients at Aga Khan Hospital Dar es salaam from January to June 2019. Patient’s medical records were reviewed and data was collected for analysis of venous thromboembolism assessment and compliance with Caprini risk assessment model. The data was entered into statistical package for the social sciences (SPSS) 25 and categorized into risk groups, frequency of patients' demographic and clinical characteristics data was calculated and the main study outcomes were analyzed with Fisher´s exact test or Pearson chi-square test for categorical variables and student t-test for continuous variables. Regression analyses were done to identify significant risk factors where by P ≤ 0.05 was considered statistically significant.

**Results:**

compliance of venous thromboembolism assessment among medical and surgical patients was similar at 78% and 80%, respectively, with a baseline 22% of all admitted patients considered at risk of venous thromboembolism, hence needing thromboprophylaxis following the Caprini risk assessment modelscore. Thromboprophylaxis practices was identified at just 25% of at-risk individuals received pharmacological prophylaxis with enoxaparin; the most commonly used agent (92%). Identified risk factors for venous thromboembolism were advancing age (>60 years), history of prior major surgery, Major surgery lasting > 60 minutes, obesity, and immobilization.

**Conclusion:**

risk assessment for venous thromboembolism should be emphasized upon admission of both surgical and medical patients. Adequate thromboprophylaxis should be prescribed upon identification of patients at risk.

## Introduction

Venous thromboembolism (VTE) is a common complication among admitted medical and surgical patients. Venous thromboembolism (VTE) is often used for a condition which includes pulmonary embolism (PE) and deep venous thrombosis (DVT). The reported incidence ranges from 10% to 40%, in patients who are not receiving appropriate thromboprophylaxis. Orthopaedic patients particularly are at higher risk of developing VTE which is associated with both mortality and morbidity. Pulmonary embolism is associated with approximately 10% of inpatient hospital deaths. Morbidities includes recurrent thrombosis, pulmonary hypertension and post thrombotic syndrome [1]. Hereditary and acquired conditions, medical and surgical illnesses, and other predisposing factors have been identified as causes of VTE. Accumulation of multiple risk factors increases the risk of developing VTE. The common risk factors which predispose to VTE includes but are not limited to acute medical illness, surgery (especially pelvic or orthopaedic), immobility, malignancy, hormone replacement therapy or the oral contraceptive pill, inherited thrombophilia and obesity [2]. Upon admission accurate patient risk factor assessment so as to identify individuals who are at risk of VTE is critical to improve compliance with thromboprophylaxis guidelines. To identify medical and surgical patients at risk of venous thromboembolism, a Caprini risk assessment model (RAM) has been developed by University of Michigan health system [3]. Because it is categorical and relatively easy to estimate, the Caprini ram has been widely used in hospitalized patients for risk assessment [4-6].

There is a well-established evidence that thromboprophylaxis prevents venous thromboembolism, due to its cost effectiveness, efficacy and risk-benefit ratio. International guidelines recommend that surgical and medical patients are evaluated upon admission for VTE risk and appropriate prophylaxis should be initiated for patient at moderate or high risk [6-9]. The Joint Commission for International Accreditation have also introduced VTE prophylaxis as one of the International hospital inpatient quality measures in hospitalized medical and surgical patients. This measures physicians practices towards complying with thromboprophylaxis practices and document the reasons if thromboprophylaxis is omitted [10]. Despite well-established evidence that thromboprophylaxis reduces VTE incidence, many studies have shown that thromboprophylaxis for patients at risk of VTE is underutilized [11-14]. The Aga khan Hospital Dar es Salaam has VTE risk assessment tools (based on Caprini RAM) in place to assess patients´ VTE risk upon admission and provide appropriate thromboprophylaxis. Primary thromboprophylaxis compliance for patients at risk of VTE provides the most significant opportunity to prevent VTE to enhance quality patient care and safety in the hospital. The actual incidence of medical and surgical patients at risk for VTE and compliance to thromboprophylaxis admitted at our hospital is currently not known. The aim to conduct this study was to determine the baseline prevalence of hospitalized medical and surgical patients at risk of VTE and whether they received appropriate thromboprophylaxis in accordance with 8^th^ ACCP Guidelines as well as determine the common risk factors for VTE.

## Methods

**Study setting and population:** this was a single-centre retrospective study of patients´ medical records from the medical and surgical department of the Aga Khan Hospital, Dar es Salaam. Compliance with VTE risk assessment for patients admitted from January 2019 to June 2019. We included all adult medical and surgical patient admitted in hospital during the study period. The patients with pre-established DVT or PE upon admission and those with contraindications to pharmacological thromboprophylaxis were excluded.

**Data collection and analysis:** a printed data collection form was used to collect data from medical record files, patients´ demographics, admission details, assessment of VTE risk based off the Caprini RAM, identified risk factors for VTE, the use of pharmacological VTE prophylaxis as per 8^th^ ACCP guidelines and choice of prophylaxis. The Caprini ram tool used was the updated 2013 version [15].

The sample size was calculated based on Slovin´s formula


$$n=\frac{N}{1+Ne^{2}}$$

whereby “n” represented sample size, “P” represented population size and “e” represented margin of error [16]. Confidence interval was kept at 95% with a margin of error of 5% thus with an estimated total number of patients of 1000 a sample size of 286 participants was calculated.

Data was entered and analyzed by the primary researcher into statistical package for the social sciences (SPSS) 25 whereby patients risk assessment data recorded as per Caprini ram was categorized into risk groups of very low, low, moderate and high. Frequency of patients' demographic and clinical characteristics data was recorded and the main study outcomes was analyzed with Fisher´s exact test or Pearson chi-square test for categorical variables and student t-test for continuous variables. Regression analyses were done to identify significant risk factors where by P≤ 0.05 was considered statistically significant.

**Ethical considerations:** the study population was not placed at additional risk with no interference with patient care. Permission to carry out the study was received from the Aga Khan University Ethical Review Board reference AKU/2020/0165/fb as well as from the hospital for use of medical records.

## Results

A total of 300 patients were included into the study of whereby 111 (37%) were surgical patients who met the inclusion criteria and 189 (63%) were medical patients. It included 176 (59%) males and 124 (41%) females at a male to female ratio of 1.42: 1 with an average age of 42 ± 16 years. Of these patients VTE risk assessment, according to the Caprini risk assessment model, was carried out on 237 (79%) of patients in which 80% of surgical patients and 78% of medical patients were assessed. Compliance of VTE assessment between medical and surgical patients has a p value of 0.383 hence p > 0.05 thus no statistical significance in difference in comparison of compliance of assessment. Of those assessed 90 (38%) were identified to be at very low risk, 96 (41%) at low risk, 36 (15%) at moderate risk and 15 (6%) at high risk of developing VTE. In accordance with the 8^th^ ACCP guidelines patients with moderate and high risk, with no contraindications to anticoagulants, should be started on pharmacological prophylaxis. A total of 51 (22%) patients were at either moderate of high risk of which 13 (25%) received thromboprophylaxis.

Of the 51 patients, 30 were surgical and 10 (33%) received prophylaxis while 21 were medical and 3 (14%) received prophylaxis. Of the 13 patients on thromboprophylaxis enoxaparin was the most commonly used prophylactic drug accounting for 12 (92%) cases. Table 1 summarizes the assessment and thromboprophylaxis practices in medical and surgical patients. From the 237 patients assessed, the risk factors that were significantly associated with an increased VTE were advancing in age of more than 60 years (p < 0.001), history of prior major surgery (p < 0.001), Major surgery lasting > 60 minutes (p < 0.001), obesity (p < 0.001) and patient immobilized at bed rest (p < 0.022). All the identified risk factor findings are summarized in Table 2. For surgical patients, major surgery, obesity and age of more than 60 years were the most common risk factors while among medical inpatients, age> 60 years and patient’s immobilization were the significant risk factors. Table 2 summarizes findings on identified VTE risk factors.

**Table 1:** venous thromboembolism risk assessment and thromboprophylaxis practice

	Surgical 111 (%)	Medical 189 (%)	Total 300 (%)
**Number of patients assessed**	88 (80%)	149 (78%)	237 (79)
Very low risk for venous thromboembolism	23 (26%)	67 (45%)	90 (38%)
Low risk for venous thromboembolism	35 (40%)	61 (41%)	96 (41%)
Moderate risk for venous thromboembolism	20 (23%)	16 (11%)	36 (15%)
High risk for venous thromboembolism	10 (11%)	5 (3%)	15 (16%)
In need for pharmacological thromboprophylaxis	30 (34%)	21 (14%)	51 (22%)
Received thromboprophylaxis	10 (33%)	3 (14%)	13 (25%)

**Table 2:** identified venous thromboembolism risk factors

Risk factor	Surgical	Medical	Percentage	Chi Square/Fisher exact value	p-value
Age 40 - 59	19	41	25.3%	0.001	0.999
Age 60 - 74	13	17	12.7%	41.46	<0.001
Age > 75	4	5	3.8%	34.12	<0.001
History of prior major surgery	19	9	11.8%	28.88	<0.001
Minor surgery planned	22	0	9.3%	0.46	0.585
Major surgery lasting > 60 minutes	10	4	5.9%	19.07	<0.001
Elective major lower extremity arthroplasty	1	0	0.4%	3.67	0.215
Obesity (BMI > 30)	14	12	11.0%	64.87	<0.001
Oral contraceptive use	3	1	1.7%	1.12	0.580
Pregnancy or post-partum (< 1 month)	1	0	0.4%	0.28	0.999
Swollen legs	5	1	2.5%	2.96	0.116
Varicose veins	2	0	0.8%	7.36	0.046
Leg plaster cast or brace	1	0	0.4%	3.67	0.215
Hip, pelvis or leg fracture (< 1 month)	1	0	0.4%	3.67	0.215
Travel history	0	1	0.4%	0.28	0.999
Bed rest	3	26	12.2%	5.27	0.022
Serious lung disease (< 1 month)	0	3	1.3%	3.67	0.118
Congestive heart failure (< 1 month)	0	3	1.3%	0.251	0.518
Sepsis (< 1 month)	1	1	0.8%	7.36	0.046
Stroke (< 1 month)	0	1	0.4%	3.67	0.215
Present cancer or chemotherapy	0	1	0.4%	3.67	0.215
Gender	88	149	100%	1.01	0.338

## Discussion

Medical and surgical inpatients both have genetic and acquired conditions which could predispose to VTE. Patients with multiple risk factors are particularly at increased risk. Common risk factors suggested from other studies include advancing age, obesity, surgery, anesthesia, immobility, malignancy, varicose veins, trauma or genetic traits linked to hypercoagulability [17,18]. Most of the aforementioned risk factors were identified in our cohort of inpatients upon assessment of VTE risks. The most common identified risk factors included advancing age, history of major surgery, obesity and patient immobilized at bed rest. Upon VTE assessment of patients 23% and 11% of surgical patients were considered to be at moderate and high risk respectively of developing VTE. Whereas 11% and 3% of medical patients were considered at moderate and high risk respectively. The findings in difference in VTE risk among medical and surgical patients were consistent with other studies which evaluated surgical patients at higher risk than medical patients including the large endorse study on VTE risk and prophylaxis [5,19]. Post-operative immobility, trauma and anesthesia time are factors that increases the risk among surgical patients.

We identified 22% of all admitted patients in need for thromboprophylaxis in accordance with the Caprini risk assessment score of which only 25% of patients received pharmacological thromboprophylaxis, of which enoxaparin was the most commonly used drug. Our findings corroborate that of earlier studies. A similar study done in Jordan that also uses the Caprini model and ACCP guidelines showed that only 26% of high risk patients received thromboprophylaxis [20]. Studies by Zobeiri in Pakistan (ref), and quratulain underscores on the underutilization of VTE prophylaxis in high risk groups as their studies revealed that only 3.2% and 3.52 % respectfully, received thromboprophylaxis [5]. Surprisingly these suboptimal levels of thromboprophylaxis exist despite good knowledge and attitude towards prophylaxis among clinicians [21]. Thromboprophylaxis practice and implementation is higher in the high income countries (HIC) however not optimal as VTE occurs significantly more among high risk patients that did not receive thromboprophylaxis despite knowledge of VTE risk [22].

Two confirmed VTE events occurred in the hospital in the defined period of study, each a PE confirmed with a computed tomography pulmonary angiogram. Both patients were identified as high risk according to the Caprini risk assessment model however neither case received thromboprophylaxis. One case developed the PE while still in the hospital while the other after discharge. Each patient ultimately ended up in the intensive care unit, with prolonged hospital stay, increased number of investigations, higher financial expenses and larger pharmacological burden on the patient. Surgical procedures increases the risk for VTE however studies show that medical patients too are at risk of developing a VTE therefore both populations should be assessed on admission and given appropriate prophylaxis [23]. Compliance rates of VTE assessment in our study among medical and surgical patients was similar with 78% and 80% of medical and surgical patients respectively assessed. However, differences in prescribing thromboprophylaxis among patients at risk of VTE exists with 33% of at-risk surgical patients receiving prophylaxis while only 14% of at risk medical patients received prophylaxis.

**Limitations:** the findings were based off data attained from an urban center that utilizes a risk assessment score hence generalizing it to a bigger multi center sample would be a limitation as most centers in the country do not utilize such scores. Another limitation is the study does not take gynecological patients into account which are a high risk for VTE populations hence could be looked into in follow up studies.

## Conclusion

Compliance of VTE risk assessment among medical and surgical patients was similar at 78% and 80% respectively with a baseline 22% of all admitted patients considered at risk in need for thromboprophylaxis in accordance with the Caprini RAM score. However, thromboprophylaxis practices was identified at just 25% of at risk individuals received pharmacological prophylaxis. Therefore, we recommend the implementation thromboprophylaxis practices as per ACCP guidelines and advocate towards training thromboprophylaxis measures into physicians' practices.

### What is known about this topic

- Hospitalized patients are at risk of venous thromboembolism;
- Risk assessment scores can be used on admission to identify patients at high risk of venous thromboembolism.

### What this study adds

- There is good compliance with scoring patients for risk of venous thromboembolism at 79% at our study setting however there is still room for improvement to meet the ACCP recommendations;
- Thromboprophylaxis practices are still suboptimal, whereby only 25% of identified patients at risk at our study setting were initiated on thromboprophylaxis, despite identification of patients at risk for venous thromboembolism via a risk assessment model;
- Identification of most common risk factors for venous thromboembolism among hospitalized patients were major surgery, obesity, age of more than 60 years and patient's immobilization.
